# Prevalence and Clinical Implications of Ascites in Gastric Cancer Patients after Curative Surgery

**DOI:** 10.3390/jcm10163557

**Published:** 2021-08-13

**Authors:** Ju-Hee Lee, Sung-Joon Kwon, Mimi Kim, Bo-Kyeong Kang

**Affiliations:** 1 Department of Surgery, Hanyang University College of Medicine, 222 Wangsimri-ro, Seongdong-gu, Seoul 04763, Korea; sjkwon@hanyang.ac.kr; 2 Department of Radiology, Hanyang University College of Medicine, 222 Wangsimri-ro, Seongdong-gu, Seoul 04763, Korea; bluefish010@naver.com (M.K.); msbbogri@naver.com (B.-K.K.)

**Keywords:** gastric cancer, ascites, postoperative follow-up

## Abstract

We aimed to determine the frequency and clinical significance of ascites that developed during the follow-up period in patients who underwent curative resection for gastric cancer. The study included 577 patients with gastric cancer who underwent curative gastrectomy. Among them, 184 showed ascites in postoperative follow-up images. Benign ascites was observed in 131 of 490 patients without recurrence, 48 patients (of 87) with recurrence had malignancy-related ascites, and the remaining 5 patients had ascites only prior to recurrence. In most patients without recurrence (97.7%) and in 50% of patients with malignancy-related ascites, the ascites was small in volume and located in the pelvic cavity at the time that it was first identified. However, with the exception of nine patients, malignancy-related pelvic ascites occurred simultaneously or after obvious recurrence. Of those nine patients who had minimal pelvic ascites before obvious recurrence, only one had a clear association with a malignancy-related ascites. In the multivariate analysis, an age of ≤45 was the only independent risk factor for the occurrence of benign ascites. A small volume of pelvic ascites fluid is common in young gastric cancer patients who do not have recurrence after gastrectomy, regardless of sex. It is rare for ascites to be the first manifestation of recurrence.

## 1. Introduction

Gastric cancer is one of the leading causes of cancer-related deaths worldwide [1] and most gastric cancer-related deaths are due to recurrence [2]. The peritoneal region is the most common site of gastric cancer recurrence and is associated with a poor prognosis [3,4,5]. Diagnosis of peritoneal metastasis is typically determined by a computed tomography [CT] scan. Ascites is one of the most common findings suggestive of peritoneal carcinomatosis; others include peritoneal thickening, nodularity, and contrast enhancement in CT [6,7,8,9]. Although there is little convincing evidence that intense surveillance improves survival, routine follow-up after curative resection for the early detection of recurrence in gastric cancer is considered general practice, as some research findings indicate that asymptomatic patients had longer post-recurrence and overall survival than symptomatic patients [10,11,12]. Physicians often encounter ascites in abdominal imaging during the post-gastrectomy follow-up period and there are concerns that this finding may indicate early peritoneal recurrence, especially in men, despite a lack of evidence otherwise. Preoperatively detected ascites in CT strongly suggests the presence of peritoneal metastasis and free cancer cells in patients with advanced gastric cancer [13,14]. However, the clinical significance of ascites detected by postoperative CT or other abdominal imaging during the follow-up period is not well-studied. In this study, we evaluated the frequency and clinical implications of ascites in patients who underwent curative surgery for gastric cancer.

## 2. Materials and Methods

A total of 634 patients with gastric cancer who underwent curative gastrectomy at the Hanyang University Seoul Hospital between January 2008 and December 2015 were selected from a prospective gastric cancer database. Fifty-seven patients were excluded for the following reasons: (1) mortality after surgery (*n* = 3); (2) synchronous or metachronous cancers (*n* = 14); (3) recurrent ascites due to liver cirrhosis or chronic kidney disease (*n* = 3); (4) ascites due to ileus (*n* = 1); and (5) follow-up loss or a short follow-up time of <12 months due to unknown causes (in patients without recurrence) after surgery (*n* = 35). A postoperative follow-up was conducted every 3–6 months for up to 5 years and annually thereafter. Standard clinical practice included evaluation by physical examination, laboratory tests including the measurement of tumor markers, radiologic imaging, and endoscopy. Imaging was conducted alternatively by abdominopelvic and chest CT and abdominal sonography. Medical records of the remaining 577 patients were retrospectively reviewed. The median period of follow-up was 61.0 months (range of 4.0–146.0 months).

**Figure 1:**
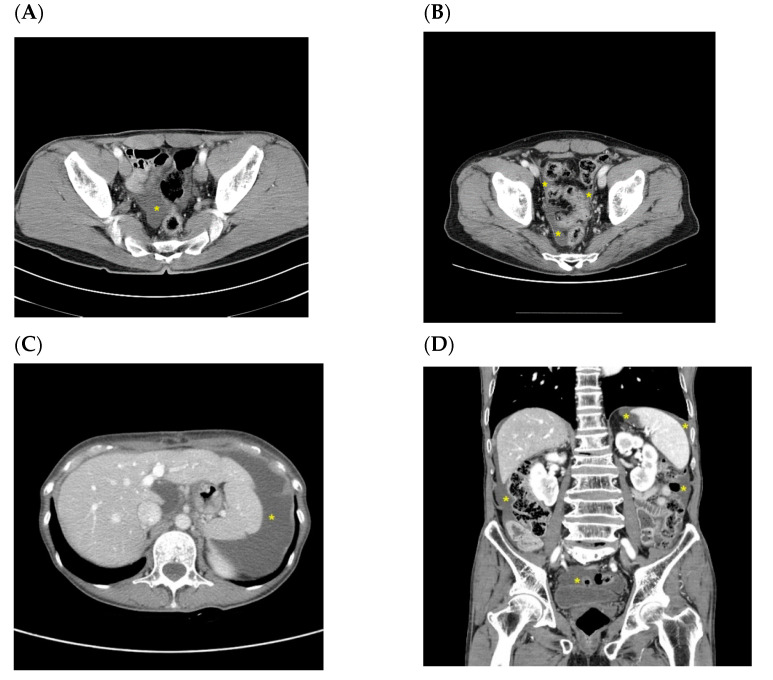

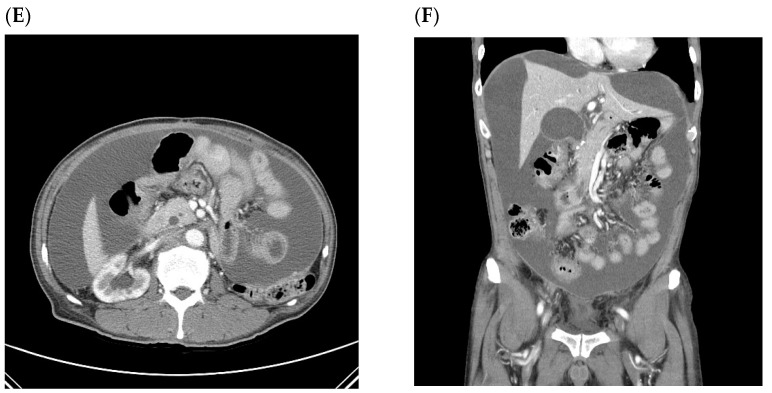
Typical ascites imaging in CT: (**A**) benign small pelvic ascites; (**B**) malignant small pelvic ascites with peritoneal thickening; (**C**) benign moderate ascites in perisplenic area (left abdomen); (**D**) malignant moderate ascites; and (**E**,**F**) alignant large ascites. Yellow stars indicate ascites.

Ascites was primarily detected in CT imaging, having been initially identified by abdominal sonography in only one patient. Images were reviewed by at least two experienced radiologists and ascites was considered present when a low radiologic density of ≤10 Hounsfield units was found within the abdominal cavity outside the intra-abdominal or pelvic organs. Intraperitoneal fluid collection that occurred within 3 months after surgery was excluded to distinguish from postoperative changes in benign ascites. The volume of ascites fluid was estimated using ruler grids applied to CT images using the method described by Chang et al. [15]. A small degree of ascites was defined as a volume of <50 mL, moderate as 50–500 mL, and large as >500 mL (Figure 1).

Cases of benign ascites were those in which patients developed ascites without recurrence during the follow-up period. None of the patients had an interval of <12 months from identification of ascites to the follow-up conclusion, excluding eight patients whose follow-up period exceeded to 5 years without recurrence. If only ascites was present without symptoms or other findings suggesting intra-abdominal recurrence, the ascites was considered benign at that time and routine radiologic follow-up was performed according to the gastric cancer follow-up protocol of our hospital. Short-term radiologic tests including positron emission tomography and/or abdominopelvic CT were performed within 3 months in 40 patients with benign ascites for the following reasons: (1) an advanced-stage disease with newly detected ascites (*n* = 13); (2) a remaining or increased infiltration around the surgical site (*n* = 11); (3) combined intra-abdominal lymphadenopathy, ultimately confirmed as reactive lymph node enlargement by repeat tests (*n* = 7); (4) combined abnormal laboratory findings (*n* = 2) or levels of tumor markers (*n* = 1); (5) complaints of abdominal symptoms (*n* = 1); (6) the presence of portal vein thrombosis; (7) moderate volume of ascites fluid (*n* = 1); (8) an increased volume of ascites fluid compared to the findings immediately after surgery (*n* = 2); and (9) a liver cyst of increasing size (*n* = 1). The phrase “malignancy-related ascites” is used as a more appropriate descriptor than “malignant ascites” considering malignant cells were confirmed by ascites cytology in only some patients with recurrence. Intra-abdominal recurrence or peritoneal metastasis was diagnosed by serial changes in the CT and/or positron emission tomography performed when recurrence was suspected based on the CT. A histological examination of biopsy specimens or ascites cytology for patients with recurrence was performed whenever possible. Death due to disease progression was confirmed in all patients classified as having recurrence.

Statistical analyses were performed using SPSS version 22.0 (SPSS Inc., Chicago, IL, USA). Chi-squared tests and independent Student’s *t*-tests were used for comparisons between groups. A binary logistic regression model was used for multivariate analysis. The threshold for statistical significance was set at *p* ≤ 0.05.

## 3. Results

**Figure 2:**
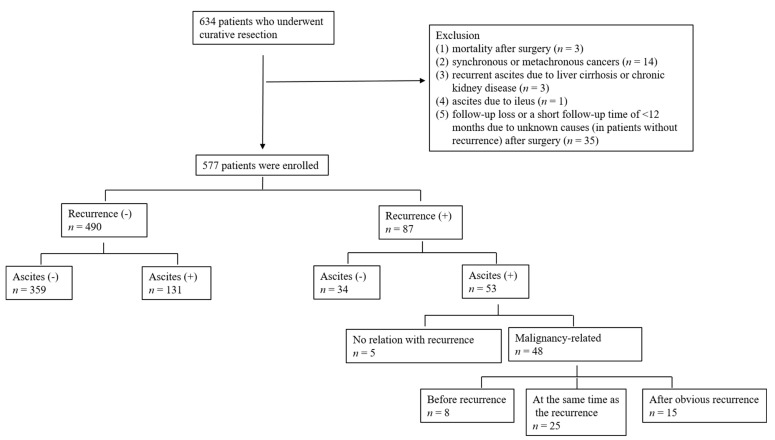
Study population.: +, presence; −, absence.

Of the 577 eligible patients, ascites was identified in 184 patients during the follow-up period. Among them, 131 patients had benign ascites, accounting for 26.7% of the 490 patients without recurrence. Of the 131 patients with benign ascites, 78 were male (78/328; 23.8% of all males without recurrence) and 53 were female (53/162; 32.7% of all females without recurrence). Ascites was observed in 53 (60.7%) of the 87 patients with recurrence. Patients fell into three groups: (1) 40 patients with malignancy-related ascites at the same time as the findings of peritoneal seeding or intra-abdominal recurrence, including one patient who on initial recurrence had ascites only; (2) eight patients with ascites presumed to be benign before obvious recurrence and considered to have malignancy-related ascites after the obvious recurrence; and (3) five patients with ascites before recurrence but no evidence of malignancy-related ascites upon recurrence. Groups (1) and (2) were classified as patients with malignancy-related ascites. Among 48 patients with malignancy-related ascites, ascites appeared prior to recurrence in eight patients (16.7%, Group (2)), simultaneous with the recurrence detection in 25 (48.1%) and following recurrence in 15 (31.3%) (Figure 2).

Comparisons between patients with and without recurrence are shown in Table 1. The T and N stages, type of resection, mean albumin and hemoglobin level, and history of adjuvant chemotherapy were significantly different between patients with and without recurrence. The most common location of ascites at first appearance was the pelvic cavity both in patients with (29/53, 54.7%) and without (128/131, 97.7%) recurrence, followed by the whole abdominal region (16/53, 30.2%), perihepatic area (6/53, 11.3%), and paracolic gutter (2/53, 3.8%) in patients with recurrence. In the remaining patients without recurrence, ascites was located in the perihepatic area (2/131, 1.5%) and the left abdominal cavity (1/131, 0.8%). Ascites fluid was small in volume and located in the pelvic cavity in most patients without recurrence (128/131, 97.7%). In the majority of patients with malignancy-related ascites, the volume of ascites fluid at first detection was small (33/48, 68.8%). Among 131 patients with benign ascites, repeatability was observed in 79 (60.3%). With the exception of one case, there was no difference in the amount and location of ascites after the initial detection in patients with recurrent benign ascites. The one exceptional case had a moderate amount of benign ascites in the perisplenic area (Figure 1C) and a small volume of pelvic ascites fluid on later examination. Median time for the first appearance of ascites was 10.5 months post-surgery (range of 3.0–108.0) in all patients with ascites, 9.0 months (range of 3.0–108.0) in those with benign ascites, and 11.5 months (range of 3.0–71.0) in those with recurrence. There was a significant difference in the mean age of males and females at the detection of benign ascites (58.8 ± 11.5 vs. 51.6 ± 12.4, *p* = 0.001). Repeatability (45 (57.0%) vs. 34 (65.4%), *p* = 0.366) and the history of adjuvant chemotherapy (32 (41.8%) vs. 18 (34.6%), *p* = 0.466) were not significantly different between males and females with benign ascites.

According to the presence or absence of ascites, sensitivity, specificity, accuracy, and positive and negative predictive values for intra-abdominal recurrence were 60.9%, 73.3%, 71.4%, 28.8%, and 91.3%, respectively. Positive and negative likelihood ratios were 2.28 and 0.53, respectively. Values for intra-abdominal recurrence were then calculated according to location of ascites (pelvic vs. other). Sensitivity, specificity, and positive and negative predictive values for the pelvic location were 54.7%, 2.3%, 18.5%, and 1.1%, respectively, and for other locations, 45.3%, 97.7%, 88.9%, and 81.5%, respectively. The positive and negative likelihood ratios for the pelvic location were 0.56 and 19.7, respectively, and 19.7 and 0.56 for other locations, respectively.

Risk factors for the occurrence of benign ascites were evaluated in recurrence-free patients (Table 2). Univariate analyses showed that younger age (≤45), a pN2–3 stage, and a history of adjuvant chemotherapy were associated with the occurrence of benign ascites. Patient sex (*p* = 0.065) and pT stage (*p* = 0.054), significant at the 0.1 level, were included with these factors in a multivariate analysis to evaluate the risk for benign ascites. Younger age (≤45) was the only independent risk factor associated with the occurrence of benign ascites post-surgery.

**Figure 3:**
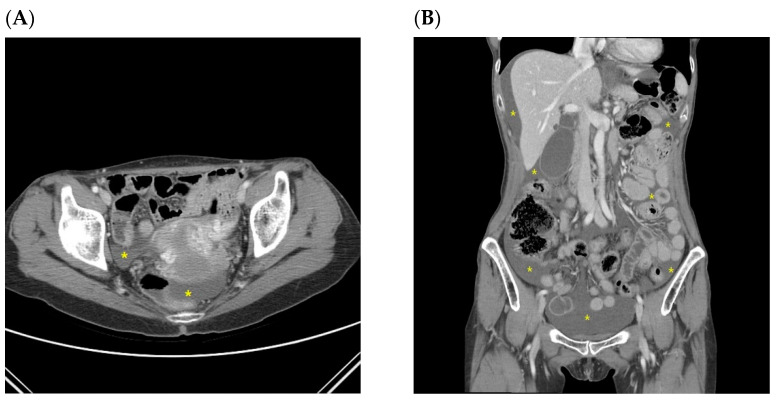
CT images of a patient on initial recurrence of malignant ascites without other CT findings related to peritoneal seeding, later confirmed by cytology. A small volume of pelvic ascites fluid was observed at first appearance (**A**). Follow-up CT (**B**) showed an increased volume of ascites fluid. Yellow stars indicate ascites.

Table 3 shows the characteristics of the nine patients with undefined ascites at the first discovery. This group includes eight patients with ascites presumed to be benign before definitive recurrence and one patient with eventual recurrence who developed ascites without other evidence of peritoneal and/or intra-abdominal recurrence. Short-term follow-up abdominopelvic CT and ascites cytology examinations were conducted one month later due to increased ascites, and peritoneal recurrence was eventually confirmed in the latter patient mentioned previously (Figure 3). All of these patients were in an advanced stage of pathology and the ascites fluid was small in volume and located in the pelvic cavity at first appearance.

## 4. Discussion

In gastric cancer patients, the most common cause of ascites after surgery is thought to be intra-abdominal recurrence but this is not supported by our study. In this study, a small volume of pelvic ascites fluid that had no identified pathological cause was noted in a substantial number of patients, notably an incidental finding during regular follow-ups. Malignancy-related ascites was observed in only 28.8% of patients with a history of ascites.

Normal peritoneal fluid that keeps the peritoneum moist and smooth may accumulate in the deep region of the pelvis in both males and females [16]. It is more frequently observed in premenopausal females than in males or postmenopausal females. In premenopausal females, the fluid that accumulates in the pelvis is thought to originate from ovarian exudation and decreased absorption of peritoneal fluid due to adhesions caused by various factors including endometriosis [17,18]. In a board sense, this fluid is called “physiologic ascites”. The precise incidence of physiologic ascites has rarely been studied. According to Yoshikawa et al., a small amount of physiologic pelvic ascites was observed in 3.8% of healthy males and 16.8% of healthy postmenopausal females in pelvic magnetic resonance imaging conducted during health screenings [17]. In our study, benign ascites was identified in 23.8% of all male patients and 32.7% of all female patients without recurrence. There was no statistically significant difference in ascites detection between the sexes on either univariate or multivariate analyses. Peritoneal fluid accumulation seems to occur more often after gastric cancer surgery regardless of sex. A possible explanation for this is that the absorption capacity of the peritoneum decreases when it is infected or injured [19]. It is assumed that ascites accumulation increases after surgery due to peritoneal injury and adhesion. This reaction may be stronger in younger patients because an age of ≤45 was the only independent factor significantly linked to the occurrence of benign ascites in our study. In addition, young female patients are more likely to have ascites due to gynecological causes but those were not investigated in this study because of insufficient medical records. Therefore, the level of concern regarding recurrence is lower if the patient’s ascites has a gynecological cause.

Cheon et al. reported that a small amount of pelvic fluid was detected in follow-up CT after curative surgery for gastric cancer in 3.9% of male patients [20]. This incidence is quite low when compared with our study. The authors obtained data only from radiology reports without a review of all CT images, whereas in our study, all images were independently reviewed by two radiologists. In addition, their definition of a “small” volume of ascites fluid was less than 20 mL, while in our study, it was defined as less than 50 mL. These reasons may explain the discrepancy between the results of the two studies. Further prospective observation is required for clarification.

According to reports from South Korea as well as western countries, malignant tumors are the second most common pathologic cause of ascites, following portal hypertension due to cirrhosis [21,22]. Malignancy-related ascites typically develops in the setting of recurrent and/or advanced cancer. The primary pathophysiological mechanism of malignancy-related ascites is peritoneal carcinomatosis, which blocks the drainage of lymphatic channels and increases vascular permeability [23]. Along with peritoneal carcinomatosis, some tumors may metastasize in the liver, which can cause ascites because of the obstruction/compression of the portal veins, further leading to portal hypertension or liver failure. Other types of tumors such as lymphomas can cause lymph node obstruction with accumulation of chylous ascites [24]. In gastric cancer, multiple pathophysiological mechanisms of ascites formation can occur but the primary mechanism is peritoneal carcinomatosis. There are few reports considering ascites as the first manifestation of gastric cancer despite its being the first detected sign of intra-abdominal malignancy in 50% of patients with peritoneal carcinomatosis [23,25]. To date, there are no reports considering ascites as the first manifestation of recurrence in postgastrectomy gastric cancer patients. In our study, most cases of malignancy-related ascites were accompanied by other findings of recurrence or were discovered during disease progression. Malignancy-related ascites is indicated as a late manifestation of intra-abdominal metastasis after gastrectomy for gastric cancer, as in the first diagnosis of gastric cancer [26]. Therefore, a small amount of pelvic ascites cannot be excluded from consideration as an early indicator of peritoneal carcinomatosis. This judgment requires caution. In our study, ascites was the only initially detected sign of intra-abdominal recurrence in one patient (Table 3; Figure 3). Furthermore, among patients with ascites presumed to be benign before obvious recurrence, the possibility of malignancy-related ascites due to peritoneal recurrence cannot be excluded because of the relatively short time between the first occurrence of ascites and the recurrence in some cases (Pt. 1, 5, 6, and 8 in Table 3). All patients mentioned above were in far-advanced stages of the disease (Table 3). The possibility of recurrence should be suspected in all gastric cancer patients with advanced disease who develop ascites even when the characteristics of the ascites are similar to those of benign ascites and there are no definitive findings suggesting peritoneal metastasis.

CT is frequently used for the postoperative surveillance of patients with gastric carcinoma. CT allows for the detection of even small amounts of ascites and provides information that is difficult to obtain in ultrasonography [16]. It would be desirable if intra-abdominal recurrence could be predicted with CT-detected ascites; however, this does not seem to be possible. The likelihood ratios were not at appropriate levels in our study. According to previous reports, malignancy-related ascites is often loculated or septated, or may be absent in typical or dependent areas such as the pelvis [8,27]. Similarly, in our study, the specificity and positive and negative predictive values for intra-abdominal recurrence were improved when calculated for ascites at locations other than the pelvis. In terms of the volume of the ascites fluid, that of >50 mL in a preoperative CT was found to be related to peritoneal metastasis in gastric cancer patients in a recently published study [13]. Additionally, in our study, malignancy-related ascites fluid volumes were larger (>50 mL are considered moderate and large amounts) than benign ascites at the time of initial detection. However, except for some patients with undefined ascites, all patients with malignancy-related ascites exhibited definitive peritoneal metastasis or intra-abdominal recurrence in the CT when ascites was first detected. Ascites alone therefore seems to be inappropriate as a diagnostic marker for intra-abdominal or peritoneal recurrence in postgastrectomy gastric cancer patients.

## 5. Conclusions

The presence of small-volume pelvic ascites fluid in follow-up images has minimal clinical significance in the majority of patients who undergo gastrectomy for gastric cancer. This phenomenon is more common in younger patients, regardless of sex. Although the presence of malignant ascites alone in the pelvic cavity can precede obvious intra-abdominal recurrence involving peritoneal seeding, ascites is more likely to be an indicator of disease progression in patients with recurrent gastric cancer.

## Figures and Tables

**Table 1:** Clinicopathologic features.

	Ascites without Recurrence	Ascites with Recurrence	*p*
	*n* (%)	*n* (%)	
Sex			0.869
Male	78 (60.3)	31 (58.5)	
Female	53 (39.7)	22 (41.5)	
Age (years ± SD)	55.9 ± 12.3	60.0 ± 12.1	0.046
Type of resection			<0.001
Partial gastrectomy	105 (80.2)	26 (49.1)	
Total gastrectomy	26 (19.8)	27 (50.9)	
Surgical approach			<0.001
Open	89 (67.9)	52 (98.1)	
Laparoscopy	42 (32.1)	1 (1.9)	
pT stage			<0.001
T1	80 (61.1)	4 (7.5)	
T2	12 (9.2)	4 (7.5)	
T3	18 (13.7)	24 (45.3)	
T4	21 (16.0)	21 (39.6)	
pN stage			<0.001
N0	80 (61.1)	8 (15.1)	
N1	23 (17.6)	5 (9.4)	
N2	19 (14.5)	7 (13.2)	
N3	9 (6.9)	33 (62.3)	
Nutritional parameters			
Albumin (g/dL ± SD)	4.2 ± 0.4	4.0 ± 0.6	<0.001
Hemoglobin (g/dL ± SD)	12.3 ± 1.4	11.4 ± 1.4	<0.001
Adjuvant chemotherapy			<0.001
No	80 (61.1)	8 (15.1)	
Yes	51 (38.9)	45 (84.9)	
Location of ascites at first appearance			<0.001
Pelvic cavity only	128 (97.7)	29 (54.7)	
Whole abdomen	0 (0)	16 (30.2)	
Other	3 (2.3)	8 (15.1)	
Volume of ascites at first appearance			<0.001
Small	130 (99.2)	38 (71.7)	
Moderate	1 (0.8)	10 (18.9)	
Large	0 (0)	5 (9.4)	
Repeatability ^†^			
No	52 (39.7)		
Yes	79 (60.3)		
Timing of first appearance (months after surgery)	9.0 (range of 3.0–108.0)	11.5 (range of 3.0–71.0)	

“Ascites with recurrence” includes patients with malignancy-related ascites (*n* = 40), patients in whom ascites before recurrence were reclassified as malignancy-related ascites at a later stage (*n* = 8), and patients with benign ascites before recurrence but with no evidence of malignancy-related ascites after recurrence (*n* = 5). Abbreviations: SD, standard deviation. ^†^ In the case of ascites without recurrence.

**Table 2:** Risk factors related to the occurrence of benign ascites in disease-free patients.

	Univariate Analysis			Multivariate Analysis		
	Ascites (−), *n* (%)	Ascites (+), *n* (%)	*p*-Value	Hazard Ratio	95% CI	*p*-Value
Sex			0.065			
Male	249 (75.9)	79 (24.1)		1		
Female	110 (67.9)	52 (32.1)		1.436	0.926–2.227	0.106
Age (years)			<0.001			
≤45	9 (32.1)	19 (67.9)		6.465	2.803–14.915	<0.001
>45	350 (75.8)	112 (24.2)		1		
Type of surgery			0.211			
Total gastrectomy	306 (74.5)	105 (25.5)				
Partial gastrectomy	53 (67.1)	26 (32.9)				
Surgical approach			0.581			
Open	252 (74.0)	89 (26.0)				
Laparoscopy	106 (71.6)	42 (28.4)				
Depth of invasion			0.054			
pT1–2	283 (75.5)	92 (24.5)		1		
pT3–4	76 (66.1)	39 (33.9)		0.889	0.435–1.819	0.748
Lymph node metastasis						
pN0–1	310 (75.1)	103 (24.9)	0.049	1		
pN2–3	49 (63.6)	28 (36.4)		1.088	0.553–2.140	0.806
Adjuvant chemotherapy			0.003			
No	268 (77)	80 (23.0)		1		
Yes	91 (64.1)	51 (35.9)		2.098	0.985–4.470	0.055

+, presence; −, absence. Abbreviation: CI, confidence interval.

**Table 3:** Characteristics of patients with temporarily undefined ascites.

	Age/Sex	Type of Operation	TN Stage	Characteristic of Ascites	Timing of Malignant Ascites	Site of First Recurrence	Interval between Surgery and Recurrence (Month)	Interval between First Appearance of Benign Ascites and Recurrence (Month)
**Patients with ascites presumed to be benign before confirmation of recurrence (*n* = 8)**								
Pt 1	65/M	PG	T3N3a	small pelvic cavity	Simultaneous with recurrence	T colon (increased ascites in the pelvic cavity)	44.4	8.1
Pt 2	53/F	TG	T4bN3b	small pelvic cavity	Simultaneous with recurrence	Peritoneum (increased ascites, peritoneal thickening, bowel obstruction, and Krukenberg tumors)	18.2	13.2
Pt 3	40/F	TG	T4aN3a	small pelvic cavity	Simultaneous with recurrence	Peritoneum (nodularity and increased ascites)	73.2	70.3
Pt 4	59/F	TG	T4aN3a	small pelvic cavity	Simultaneous with recurrence	Peritoneum (increased ascites and peritoneal thickening)	25.2	21.7
Pt 5	39/F	TG	T3N3b	small pelvic cavity	Simultaneous with recurrence	Peritoneum (Krukenberg tumors, nodularity, and increased ascites)	12.1	9
Pt 6	57/F	TG	T3N3a	small pelvic cavity	Simultaneous with recurrence	Peritoneum (bowel obstruction and increased ascites)	9.8	4.4
Pt 7	61/F	TG	T3N1	small pelvic cavity	Simultaneous with recurrence	Peritoneum (increased ascites, T colon, and mesentery LNs)	29.6	26
Pt 8	68/F	PG	T4aN2	small pelvic cavity	Simultaneous with recurrence	Peritoneum (increased ascites and peritoneal thickening)	11.7	5.3
**A patient who first recurred with malignant ascites without other CT findings related to peritoneal seeding (later confirmed by cytology) (*n* = 1)**								
Pt 9	49/F	TG	T3N3a	small pelvic cavity				

Abbreviations: PG, partial gastrectomy; TG, total gastrectomy; and CT, computed tomography.

## Data Availability

The data presented in this study are available on request from the corresponding author.
